# Immune stress impairs broiler performance by affecting hypothalamic methylation modification and intestinal 5-HT synthesis

**DOI:** 10.1016/j.psj.2025.105444

**Published:** 2025-06-17

**Authors:** Bingjian Huang, Yan Wan, Guang Li, Jian Cui, Qiuyu Jiang, Xiang Zhong, Bingkun Zhang

**Affiliations:** a State Key Laboratory of Animal Nutrition and Feeding, College of Animal Science and Technology, China Agricultural University, No. 2, Yuanmingyuan West Road, Haidian District, Beijing 100193, China; b College of Animal Science and Technology, Nanjing Agricultural University, Nanjing 210095, China

**Keywords:** Broiler, Lipopolysaccharide, Immune stress, Brain-gut axis, Methylation

## Abstract

In this experiment, a chronic immune stress model was established in broiler chickens by injecting lipopolysaccharide (LPS). The objective was to investigate the impact of immune stress on performance and intestinal mucosal barrier function in broiler chickens and identify key molecular and neuroendocrine pathways contributing to these effects. We randomly divided 160 one-day-old male white-feathered broilers into two groups: the control group (CON) and the LPS immune stress group (LPS), with 8 replicates of 10 chickens each. The LPS group received 0.5 mg/kg body weight LPS injections at 14 days of age for 10 consecutive days, while the control group received saline injections.

The results indicated that LPS injection significantly decreased broiler performance (*P* < 0.05) and increased immune organ indices and serum corticosterone levels (*P* < 0.05). The mRNA levels of *IL-1β* in the spleen, as well as *TNF-α, IL-1β*, and *FABP1* in the ileum, and hypothalamic *MCH* and *PREPRO*, showed significant upregulation (*P* < 0.05). Conversely, the mRNA levels of *CHGA, JAM2, PCNA, BCL2*, and *mTOR* were significantly decreased in the ileum, along with a notable decrease in hypothalamic *NPY* (*P* < 0.05). There was also a tendency toward lower expression of mTOR protein in the ileum (*P* = 0.054). Moreover, serum 5-hydroxytryptamine (5-HT) levels and gene expression of 5-HT receptors in the ileum were significantly reduced (*P* < 0.05). Additionally, there was a significant increase in m^6^A methylation in both the hypothalamus and ileum (*P* < 0.05), affecting genes involved in neurotransmitter metabolism and signaling in the hypothalamus.

Collectively, these findings suggest that LPS-induced immune stress inhibits the mTOR/P70S6K pathway in the ileum, suppresses intestinal 5-HT synthesis, and impairs intestinal mucosal barrier function. Immune stress also affects m^6^A RNA methylation and appetite-related gene expression in the hypothalamus, leading to reduced broiler performance.

## Introduction

Immunological stress occurs when animals experience frequent infections from pathogenic microorganisms, typically due to unsanitary housing conditions. This chronic exposure activates the immune system, triggering non-specific adaptive responses against invading pathogens. In modern poultry production, broilers are particularly susceptible to immunological stress due to intensive vaccination protocols and suboptimal breeding environments. This stress induces metabolic disorders, depression, reduced appetite, and growth retardation in broilers, resulting in substantial economic losses for the poultry industry (Li et al., 2020).

Exogenous lipopolysaccharide (LPS) injection is a commonly used, stable, and reproducible method for inducing immunological stress in research studies. Current models employ doses of 0.5-3 mg/kg body weight. A single high-dose LPS injection provides rapid induction of acute immunological stress with short duration, while repeated LPS injections induce chronic immune stress similar to commercial production conditions. Immunological stress decreases thyroid hormone levels, delays gastric emptying, and reduces voluntary feed intake (Kravchenko and Zakharchenko, 2023; Hu and Guo, 2008).

The hypothalamus serves as the primary control center for regulating food intake and energy balance by integrating signals from the brain, peripheral circulation, and gastrointestinal tract (Kalra, 1997). The hypothalamus stimulates pituitary ACTH secretion and regulates HPA axis activity through corticotropin-releasing hormone (CRH) release, playing a crucial role in homeostatic appetite control. Neuropeptide Y (NPY) and agouti-related protein (AgRP) stimulate appetite, while pro-opiomelanocortin (POMC) and melanocortin-4 receptor (MC4R) inhibit appetite (Valassi et al., 2008; Kalra and Kalra, 2004; Kawakami et al., 2000; Yu et al., 2009). The neurotransmitter serotonin (5-HT) regulates feeding behavior and energy metabolism through receptor activation, affecting animal growth and production (Berthoud, 2002; Bluher et al., 2023; He et al., 2019; Martin et al., 2017; Tan et al., 2019). Additionally, 5-HT controls gastrointestinal peristalsis and secretion, as well as central neuronal mood and emotional responses. Studies demonstrate that 5-HT levels change under stress conditions (Laaris et al., 1999). N6-methyladenosine (m^6^A) represents the most prevalent eukaryotic mRNA modification, regulating multiple mRNA processing steps including splicing, export, translation, and degradation, thereby fine-tuning gene expression. Research indicates that m^6^A plays regulatory roles in acute CNS injuries, chronic neurodegeneration, brain cancer, and neuropsychiatric diseases (Chokkalla et al., 2020).

Studies demonstrate that chronic stress affects m^6^A levels in animals. Acute restraint stress in mice caused m^6^A hypomethylation in the prefrontal cortex and hypermethylation in the amygdala (Engel et al., 2018). Liu et al. found that heat stress altered mRNA methylation modification patterns in sheep liver (Lu et al., 2021). However, research on stress effects on m^6^A modification in chickens remains limited. Whether m6A modification changes under stress and whether m^6^A regulates appetite in the chicken hypothalamus remain unclear.

This study employed a controlled experimental model to induce chronic immunological stress in broiler chickens through sustained administration of low-dose LPS. Subsequently, we investigated m^6^A methylation modifications within the hypothalamus, quantified the expression levels of hypothalamic neuropeptides, and assessed ileal serotonin (5-HT) biosynthesis. The objective of these analyses was to elucidate the potential mechanistic involvement of these molecular pathways in the immune stress-mediated decline in production performance parameters.

## Materials and Methods

### Animals and treatment design

The animal experiment was conducted in Zhuozhou city, Hebei Province, China. The experimental procedures were approved by the Animal Welfare and Ethical Committee of China Agriculture University. The ethics committee number is AW02205202-01-09.

A total of 160 one-day-old male broilers (Arbor Acres) were obtained from a commercial hatchery and randomly assigned to two treatments (CON and STR) with 8 replicates of 10 broilers each. From 1 to 24 days of age, the broilers were housed according to commercial management practices. They were provided with ad libitum access to feed and water during this period. The composition of the basal diet and nutrient levels can be found in Table 1.

**Table 1:** Composition of diets and nutrient levels.

Ingredients/%	0-24 d	Nutrients	0-24 d
Corn GB2	53.84	L metabolisable energy(MJ/kg)	12.34
Soybean meal GB2	36.89	Crude protein/%	21.00
Corn gluten meal	1.00	Lysine/%	1.15
Soya bean oil	4.00	Methionine/%	0.56
Acidifier	0.30	Methionine and cystine mixture/%	0.93
Choline chloride	0.30	Threonine/%	0.88
Vitamin premixes^1^	0.03	Throsine/%	0.25
Trace element premixes^2^	0.20	Calcium/%	1.00
Talcum powder	1.22		
Calcium hydrogenphosphate	1.91		
DL-Methionine	0.27		
L-Lysine hydrochloride	0.04		
Total	100.00		

1. Vitamin premixes provide following per kg diet: VA 9500 IU, VD3 62.5ug, VE 30IU, VK3 2.65 mg, VB1 2 mg, VB6 6 mg, VB12 0.025 mg, per kg of compound feed.

2. The trace mineral premix provides following per kg diet: Copper 8 mg, Zinc 75 mg, Iron 80 mg, Manganese 100 mg, Selenium 0.15 mg, Iodine 0.35 mg.

At 14-24 days of age, the chickens in the STR group were intraperitoneally injected with LPS (Sigma–Aldrich Inc., USA) at a dosage of 0.5mg/kg body weight, while the CON group received injections of sterile normal saline.

The broiler chickens were housed in wire cages and subjected to a light-dark cycle of 23 hours of light and 1 hour of darkness throughout the experiment, following 24 hours of continuous light upon arrival. The room temperature was initially maintained at 32°C to 34°C for the first 5 days and then gradually decreased by 2°C per week until reaching a final temperature of 22°C to 24°C.

### Measurement of growth performance and organ index

Body weight (BW), feed intake (FI), and mortality were recorded on days 0, 14, 16, 18, 20, 22, and 24. Average body weight gain (BWG), average FI, and feed conversion ratio (FCR) were calculated during this trial. All performance parameters were adjusted for mortality.

On day 21, four hours after the LPS injection, eight broilers from each treatment (one broiler from each cage) were euthanized humanely and tissue samples were collected. The spleen, bursa, and thymus, which are immune organs, were collected and weighed. The organ index was calculated as organ weight (mg) divided by body weight (g).

### Determination of serum corticosterone levels by ELISA

At 21 d, 8 broilers in each treatment (1 broiler from each cage) were selected to collect blood into an ordinary serum tube. The tube was then placed at room temperature for 30 minutes, centrifuged at 3,000 g for 10 minutes, and the serum was separated and stored at −80°C until further analysis. The levels of serum corticosterone were determined according to the Elisa kit instruction. The kit used for serum corticosterone detection is a product of Shanghai Enzyme Linked Company.

### Total RNA extraction, reverse transcription, and quantitative real-time PCR

Total RNA was extracted from the hypothalamus, spleen, and ileum using Trizol reagent, following the manufacturer's instructions. The total RNA was then transcribed to obtain complementary DNA (cDNA) samples. The reverse transcription kit is the RevertAid™ First Strand cDNA Synthesis Kit manufactured by Genstar, and the real-time fluorescence quantification kit is the SYBR Premix EX TaqTM II manufactured by Genstar. The primers used were synthesized by Sangon Biotech Co., Ltd., Shanghai, China. The primer sequences are presented in Table 2.

**Table 2:** Primers of target genes for RT-PCR.

Target gene	Login number		Primer sequence (5′−3′)
*TNF-α*	*NM_204267.1*	F	CCGCCCAGTTCAGATGAGTTGC
		R	GCCACCACACGACAGCCAAG
*IL-1β*	*NM_204524.1*	F	ACTGGGCATCAAGGGCTA
		R	GGTAGAAGATGAAGCGGGTC
*OLFM*	*NM_001040463.2*	F	AGCACACCTCCCCAAAGTTC
		R	TGCAAGAGCGTTGTGGCTAT
*CHGA*	*XM_040673024.2*	F	AGGGAGTGGGAAGACTCCAA
		R	CTTGTGGGACCTGAATGCCA
*LYZ*	*NM_205281.2*	F	TGTGCCGCAAAATTCGAGAG
		R	TTCCGTAGTCGGTACTCCCA
*SI*	*XM_015291762.4*	F	GAGGCCACGTTAAGAAGGCT
		R	CACTTGCTCATGGGGGACTT
*PCNA*	*NM_204170.3*	F	GACAATGCGGATACGTTGGC
		R	TCACCAATGTGGCTGAGGTC
*LGR5*	*XM_046909876.1*	F	ACGTCTTGCAGGAAATGGCT
		R	TTGGCATCCAGGCGTAGAGA
*BCL2*	*NM_205339.2*	F	GAGTTCGGCGGCGTGATGTG
		R	CTCGGTCATCCAGGTGGCAATG
*MUC2*	*NM_001318434.1*	F	CCCTCACCCAGCCCGACTTC
		R	GCCGTTGGTGGAGGTGTTACAG
*JAM2*	*XM_046907882.1*	F	AGCCTCAAATGGGATTGGATT
		R	CATCAACTTGCATTCGCTTCA
*IFN-α*	*NM_205149.2*	F	AAGTCATAGCGGCACATCAAAC
		R	CTGGAATCTCATGTCGTTCATCG
*COX2*	*NM_001167718.2*	F	TGTCAGATCCCCTCTGCGTT
		R	ACGTGAAGAATTCCGGTGTTG
*mTOR*	*XM_040689168.2*	F	AGAACGCCGTGAGATCATCC
		R	TATGGGCCAAAGCCAGTCTC
*METTL14*	*NM_001031148.2*	F	ATTCGACCAGGATGGCTGAC
		R	GACTTGGGTGGTGGTGACTT

Target gene	Login number		Primer sequence (5′−3′)
*METTL3*	*XM_040655036.2*	F	AAGGAACACTGCCTGGTCG
		R	GGCGTTCGATCATCCCGTAG
*FTO*	*XM_040707054.2*	F	CTCCGTCCTAGCACTCCACT
		R	TACAACTCCCATCAGGCAGC
*NPY*	*NM_205473.2*	F	GATCCCGGTTTGAAGACCCT
		R	ATGCACTGGGAATGACGCTAT
*POMC*	*NM_001398117.1*	F	GCAGCTCCTCCGCAGTT
		R	ACTTGCTGTTCTCCCAGCAT
*CB1*	*XM_046914332.1*	F	GGCTGTTTCCTTATGCCCCT
		R	GCTTGGCCTTTTGTGCTACC
*CCK*	*XM_046910595.1*	F	CAGCAGAGCCTGACAGAACC
		R	AGAGAACCTCCCAGTGGAACC
*MEL1C*	*NM_205361.2*	F	CGATCACAGTGGTGGTTGTC
		R	GTCTCACCCGGTGTTTGACT
*MCH*	*XM_046907511.1*	F	AAGAGAGGAGCGATGCCTT
		R	TTTCTGCCCACACTGATTGG
*CRH*	*NM_001123031.1*	F	CTCCCTGGACCTGACTTTCC
		R	TGTTGCTGTGGGCTTGCT
*PREPRO*	*NM_204185.3*	F	AGGAGCACGCTGAGAAGGA
		R	TGCGGGCTACCGTTTATTGT
*MAOA*	*XM_046907920.1*	F	GCCTACTTCCCACCTGGCATAA
		R	GCTGCTCTCTCTCCTGCCTGTA
*5HTR1A*	*NM_001170528.1*	F	AGAACACGGAGGCCAAGC
		R	ACGGCAACCAGCAGAGGA
*5HTR2C*	*XM_046916516.1*	F	ACTTTGTCCTCATTGGGTCTTTCA
		R	TCTGTGTTGTTTTCTTTCTTGAGGC
*SERT*	*XM_046929967.1*	F	CCAAGACGCCCTGGTTAC
		R	GGCAGGCATGTTCGCAAT
*5HTR1D*	*XM_040690105.2*	F	TCGCTTACACTGTCACCCAC
		R	AGTCCGGCGTTTGGCATATT
*5HTR1B*	*XM_046914126.1*	F	GAACACGGACCACGTCCTCTACA
		R	GCTTTCTTTGGCGTCTGCTTC
*β-ACTIN*	*NM_205518.1*	F	ACTCTGGTGATGGTGTTAC
		R	GCTGTGATCTCCTTCTG

Table 3.

**Table 3:** Effect of immune stress on production performance.

Group	1-13 d			14-23 d		
	BWG(g/per/d)	FI(g/per/d)	FCR	BWG(g/per/d)	FI(g/per/d)	FCR
CON	31.02±1.096	38.55±1.265	1.24±0.026	50.76±1.967	72.07±3.328	1.41±0.060
LPS	30.74±0.454	37.77±0.689	1.23±0.016	47.69±2.510	70.79±4.726	1.50±0.083
*P-value*	0.515	0.149	0.208	0.026	0.592	0.054

### Determination of serum 5-HT levels by HPLC-UV

To prepare a stock solution of 1000 μg/mL serotonin hydrochloride, the stock solution was subsequently diluted into 6 calibrators with concentrations of 250 μg/mL, 125 μg/mL, 50 μg/mL, 20 μg/mL, 10 μg/mL, and 5 μg/mL, respectively. To the serum samples, 600 μl of 4 % perchloric acid solution was added. After 30 seconds of vortexing, the samples were sonicated in an ice water bath for 15 minutes. They were then centrifuged at 9600 rpm for 10 minutes at 4 °C, and the supernatant was filtered on a 0.2 μm diameter Nylon filtration membrane for HPLC detection.

The HPLC experiment was conducted using an Agilent HPLC instrument (Agilent Technologies, USA) equipped with an on-line Degasser Agilent 1260 Infinity, Agilent 1260 Bin pump, 1260 ALS automatic sample introduction system, and an Agilent 1290 Thermostat temperature controller. For the detection of 5-HT, the equipment was connected to an Agilent 1260 DAD VL UV diode array detector. The chromatographic separation of 5-HT was achieved using an Agilent Zorbax Extend C18 Column (4.6 × 250 mm, 1.8 μm) under isocratic elution. The mobile phase consisted of 0.1 % methanoic acid/methanol (93:7, V/V), and the wavelength of the UV detector was set at 280 nm.

### m^6^A methylation detection

Hypothalamic tissues were rapidly frozen in liquid nitrogen immediately after collection and stored at −80°C for subsequent epigenetic and transcriptomic analyses. Total RNA was extracted, fragmented, and subjected to RNA immunoprecipitation (RIP) using an m^6^A-specific antibody to enrich methylated RNA fragments. Subsequently, MeRIP-seq libraries were constructed using the NEBNext Ultra RNA Library Prep Kit and sequenced on an Illumina NovaSeq 6000 platform (paired-end 150 bp reads). Raw reads underwent quality control with FastQC, followed by alignment to the chicken reference genome (Galgal6) using Bowtie2. m^6^A-modified peaks were identified using MACS2 (*q*-value <0.05), and differential methylation analysis (fold change ≥2, *P* < 0.05) was performed with exomePeak2 to compare stress and control groups. Conserved motifs in differentially methylated regions (DMRs) were detected using HOMER, revealing a prominent GGAC motif (*P* < 1e−100). Differentially methylated genes were functionally annotated for Gene Ontology (GO) biological processes and KEGG pathways using DAVID (FDR ≤0.05), highlighting enrichment in neurodevelopmental (Wnt, mTOR, MAPK signaling) and metabolic pathways.

### Statistical analysis

All data were expressed as means±SD and analyzed using SAS 9.4 with the independent sample t-test. Differences were considered significant at *P* < 0.05. However, if the data suggested a trend, probability values ranging from 0.05 ≤ *P* < 0.10 were also mentioned in the text.

## Result

### Growth performance

**Figure 1:**
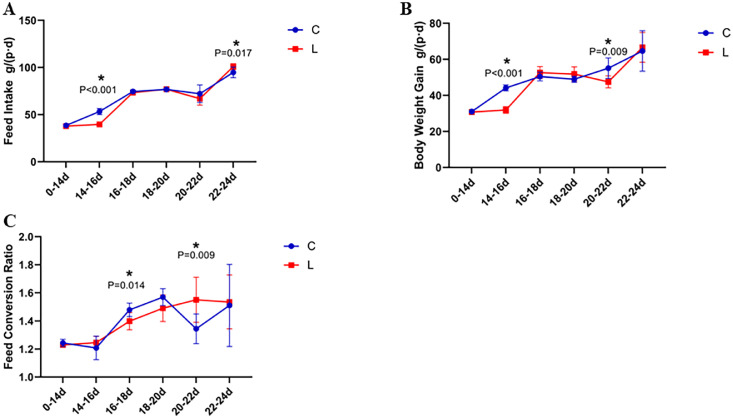
Effect of immune stress on broiler stage growth performance.

Immune stress significantly impaired growth performance, with reduced body weight gain (BWG) and a trend toward elevated feed conversion ratios (FCR) (*P* < 0.05; Figure 1). Notably, BWG declined sharply during days 14–16, with reduced feed intake persisting through days 20–22.

### Immune organ index and expression of inflammatory cytokines

The study reveals that immunological stress induced by LPS increases the immune organ index of broilers(Table 4). At 24 hours after the 6th LPS injection, the thymus index of broilers was significantly higher (*P* < 0.05), and there was a tendency for the spleen index to increase (*P* = 0.075). At 4 hours after the 7th injection, both the spleen index and bursa index were significantly higher (*P* < 0.05), and there was a tendency for the thymus index to increase (*P* = 0.054). Moreover, at 24 hours after the 7th injection, the spleen index was significantly higher (*P* < 0.05). Interestingly, the bursa index significantly increased, while the thymus index significantly decreased (*P* < 0.05) at 4 hours after the 10th injection, and the bursa index significantly increased (*P* < 0.05) at 24 hours after the 10th injection.

**Table 4:** Effect of immune stress on organ index.

Time	Organ(g/kg)	CON	LPS	*P-value*
4 h after the 6th injection	Spleen	1.02±0.206	1.20±0.329	0.202
	Bursa	1.79±0.572	1.71±0.301	0.739
	Thymus	1.76±0.284	2.05±0.329	0.074
24 h after the 6th injection	Spleen	0.98±0.278	1.28±0.347	0.075
	Bursa	1.59±0.347	1.33±0.547	0.273
	Thymus	1.16±0.174	1.60±0.450	0.021
4 h after the 7th injection	Spleen	1.07±0.276	1.61±0.343	0.003
	Bursa	1.31±0.245	1.63±0.275	0.027
	Thymus	1.43±0.133	1.93±0.612	0.054
24 h after the 7th injection	Spleen	0.83±0.165	1.33±0.262	0.001
	Bursa	2.27±0.622	2.33±0.569	0.858
	Thymus	1.28±0.335	1.30±0.219	0.879
4 h after the 10th injection	Spleen	1.31±0.216	1.22±0.170	0.355
	Bursa	1.37±0.178	1.60±0.182	0.024
	Thymus	1.43±0.303	1.12±0.209	0.033
24 h after the 10th injection	Spleen	1.13±0.225	1.26±0.265	0.321
	Bursa	1.39±0.325	1.94±0.492	0.019
	Thymus	1.27±0.305	1.08±0.262	0.209

**Figure 2:**
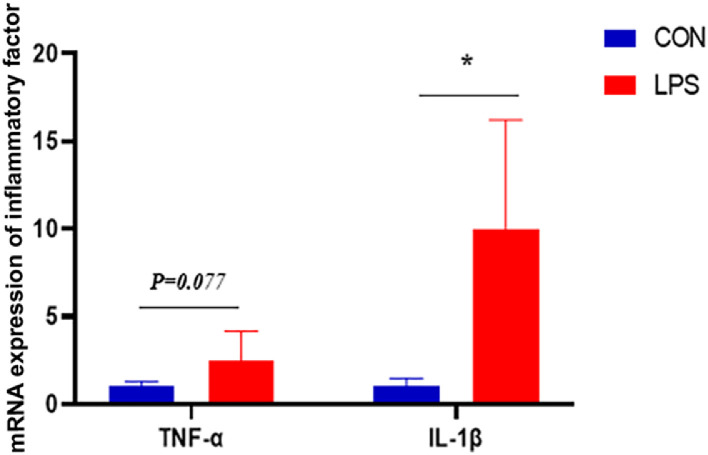
Effect of immune stress on gene expression of inflammatory factors in broiler spleen.

The effects of immunological stress induced by LPS on the expression of IL-1β and TNF-α in the spleen of broiler chickens are shown in Figure 2. The gene expression of IL-1β was significantly higher (*P* < 0.05), and there was a trend towards higher gene expression of TNF-α (*P* = 0.077) in the spleen 4 hours after the 7th LPS injection.

### Serum corticosterone levels

**Figure 3:**
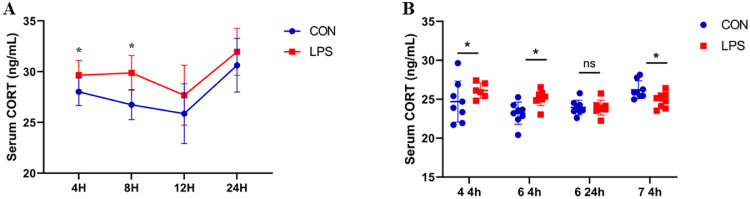
Effect of immune stress on serum corticosterone levels in broiler.

As depicted in Figure 3A, the serum corticosterone levels in broilers were significantly increased (*P* < 0.05) at 4 and 8 hours after the first injection of LPS, while there was no significant difference (*P* > 0.05) at 12 and 24 hours. As shown in Figure 3B, the serum corticosterone levels in broilers were significantly increased (*P* < 0.05) at 4 hours after the 4th and 6th injections.

### Expression of ileal genes and proteins

**Figure 4:**
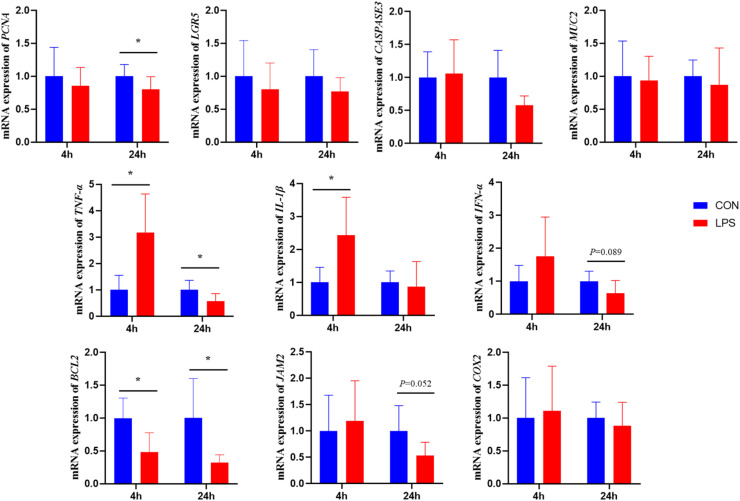
Effect of immune stress on mRNA expression of ileal genes.

As shown in Figure 4. Four hours after the 6th LPS injection, there was a significant reduction in BCL2 gene expression, and a significant increase in TNF-α and IL-1β expression (*P* < 0.05). At 24 hours after the injection, there was a significant decrease in PCNA and BCL2 gene expression (*P* < 0.05), and a tendency for JAM2 gene expression to decrease (*P* = 0.052).

**Figure 5:**
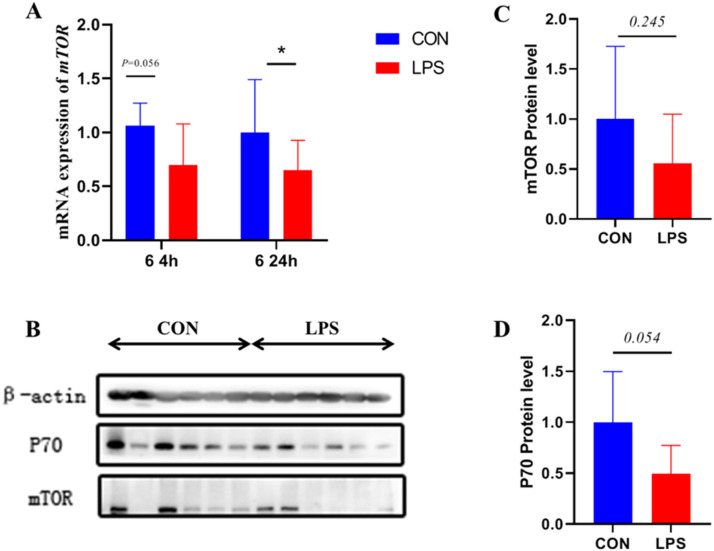
Effect of immune stress on gene and protein expression of mTOR pathway in ileum.

In Figure 5A. The expression of mTOR gene in the ileum is shown, while Figure 5C and D present the statistical data for the grey scale values of mTOR and P70S6K proteins. In the LPS group, the mRNA level of mTOR in the ileum was significantly lower (*P* < 0.05), and there was a trend towards lower mTOR protein expression in the ileum (*P* = 0.054).

### Expression of hypothalamic neurotransmitter genes

**Figure 6:**
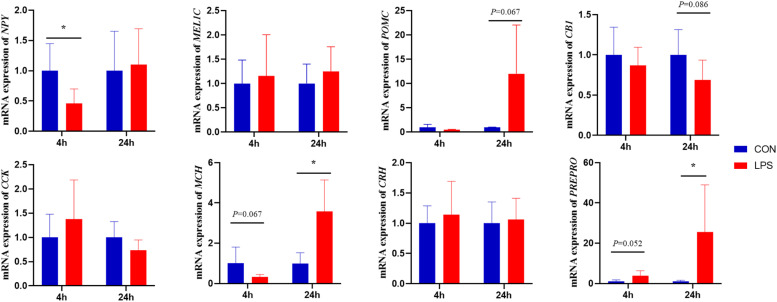
Effect of immune stress on hypothalamic neurotransmitter-related genes.

As shown in Figure 6. Four hours after the 6th LPS injection, there was a significant reduction in NPY gene expression in the hypothalamus (*P* < 0.05), and a tendency to increase the expression of the orexin precursor gene (PREPRO) (*P* = 0.052). Twenty-four hours after the 6th LPS injection, there was a significant increase in the expression of the melanin-aggregating hormone gene (MCH) and the PREPRO gene (*P* < 0.05), and a trend towards increased gene expression of former opiomelanocortin (POMC) (*P* = 0.067) and cannabinoid receptor 1 (CB1) (*P* = 0.086).

### Morphological structure of the ileum

**Figure 7:**
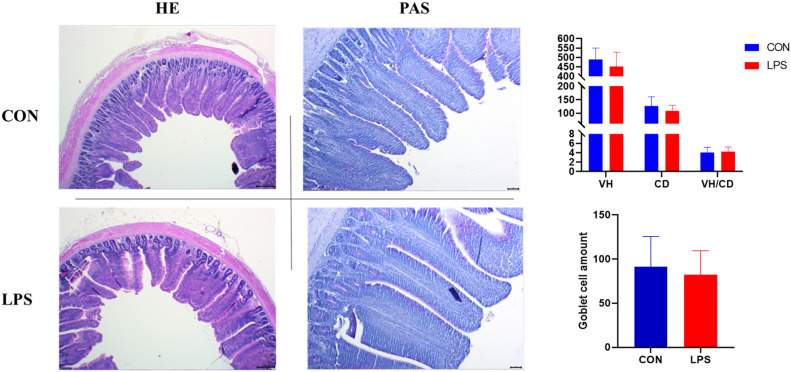
Effect of immune stress on morphological indices of broiler ileum(HE staining, 40X; PAS staining, 100X).

None of the morphological indices of the broiler ileum were significantly impacted by immune stress(Figure 7).

### Serum 5-HT levels and related gene expression in the hypothalamus and gyrus

**Figure 8:**
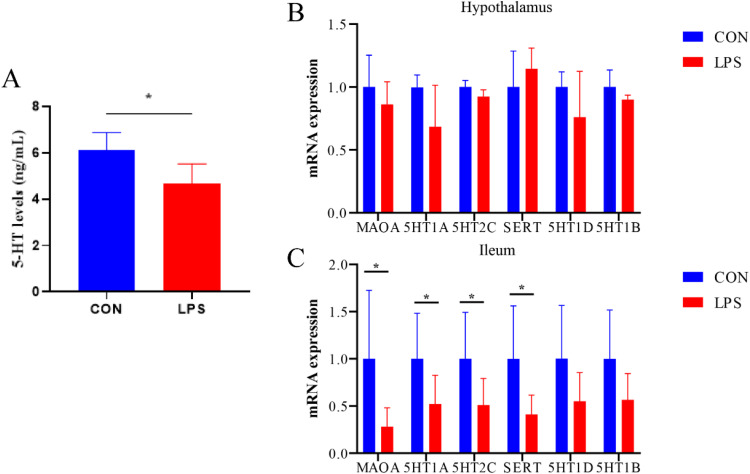
Effect of immune stress on 5-HT synthesis.

As shown in Figure 8. The levels of 5-HT in the serum of broiler chickens were significantly reduced (*P* < 0.05) under immune stress, and the expression of genes for 5-HT-related transport vectors and enzymes was significantly decreased (*P* < 0.05) in the ileum. However, there was no significant difference observed in the hypothalamus.

### Effect of immune stress on the degree of RNA m^6^A methylation in the ileum and hypothalamus

**Figure 9:**
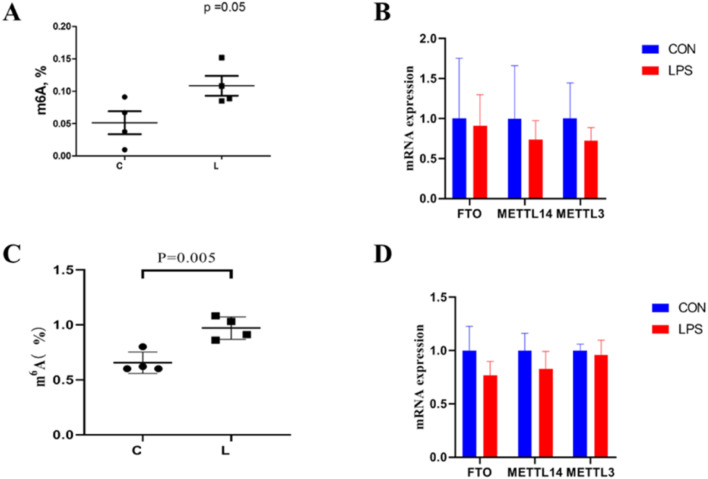
Effect of immune stress on m^6^A methylation in ileum and hypothalamus.

As shown in Figure 9. Our results indicate a significant increase in m^6^A levels in both the ileum and hypothalamus (*P* < 0.05).

**Figure 10:**
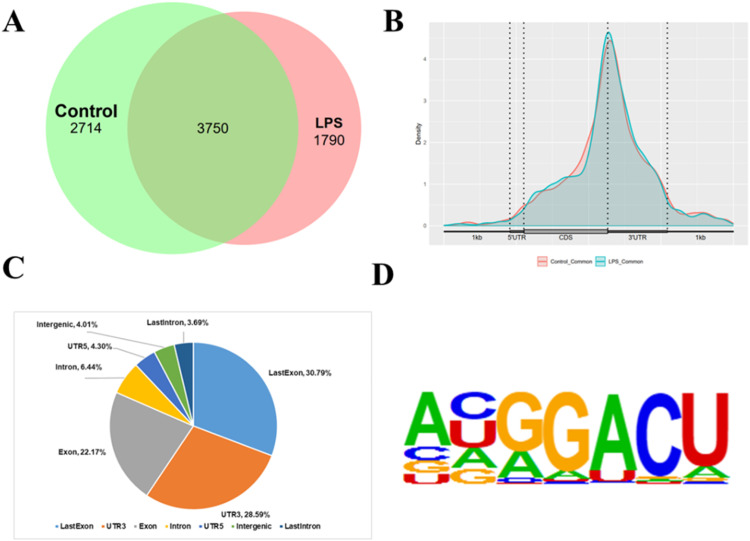
Effect of general features and topology of m^6^A modifications in the thalamus under immune stress.

A total of 6464 m^6^A-modified peaks were detected in control broilers, associated with 3625 genes. In the LPS group, 5540 m^6^A-modified peaks were detected, associated with 3918 genes(Figure 10A). Figure 10B and C reveal that m^6^A peaks are mainly distributed in the 3′UTR and Exon regions of RNA. Further analysis shows that GGAC is the motif of the base sequence of m^6^A, which is consistent with the characteristics of m^6^A modification (Figure 10D).

**Figure 11:**
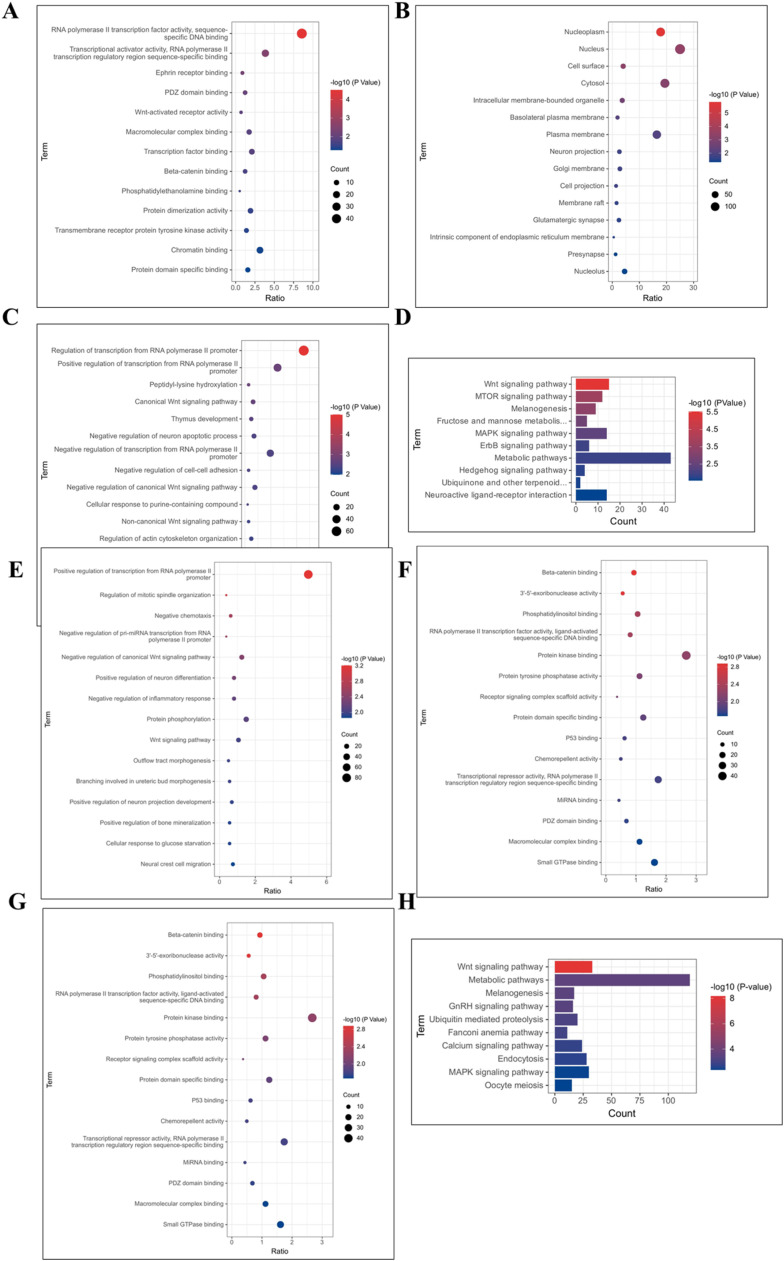

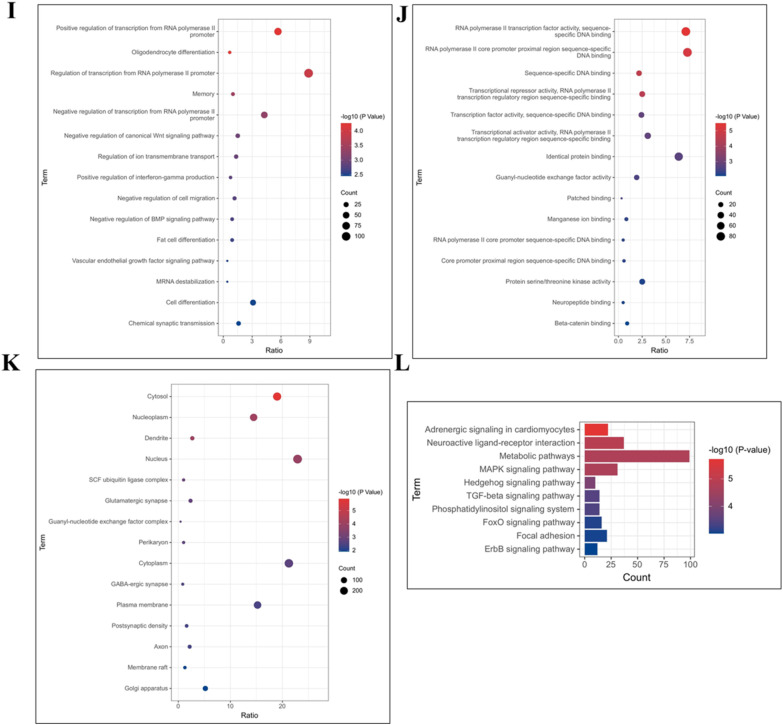
Functional analysis of immune stress on differential genes for hypothalamic m^6^A modifications.

Figure 11A-C demonstrate that genes showing differences in the enrichment of m^6^A peaks in the hypothalamus of broiler chickens in the LPS and control groups are mainly enriched in the nucleus and are involved in neuronal apoptosis, synaptic maturation, and neurological developmental processes. Figure 11D reveals that these differentially methylated modified genes are mainly enriched in classical signaling pathways for neurodevelopmental and metabolic regulation, such as the Wnt signaling pathway, the mTOR signaling pathway, fructose and mannose metabolism, the MAPK signaling pathway, and the ErbB signaling pathway. Additionally, functional enrichment analysis of methylation modification genes unique to the hypothalamus of broiler chickens in the LPS and control groups was performed in this study. Figure 11E-H show that the m^6^A modification peaks unique to the control group are mainly concentrated in the nucleoplasmic and cytoplasmic nuclei. KEGG enrichment analysis reveals that these peaks are mainly involved in the Wnt signaling pathway, metabolism, melanogenin production, and the gonadotropin signaling pathway. Figure 11I-L demonstrate that the m^6^A modification peaks unique to the LPS stress group are mainly concentrated in the cytoplasmic nucleus. These genes are also involved in the MAPK signaling pathway, adrenergic transmission, metabolism, neural interactions, and metabolism regulation signaling pathways.

## Discussion

### Construction of an immune stress model for broiler chickens through long-term intraperitoneal injection of LPS

Chronic immune stress in broiler chickens manifests as depression, reduced feed intake, altered metabolic processes, and excessive energy and nutrient expenditure required to maintain heightened immune system activity. These physiological disruptions ultimately result in accelerated skeletal muscle catabolism and growth retardation, accompanied by diminished feed conversion efficiency and compromised production performance (Ye et al., 2023; Bi et al., 2022; He et al., 2020; Liu et al., 2019). The present experimental findings corroborated these observations, demonstrating a significant reduction in average daily weight gain and a tendency toward increased feed conversion ratio in broilers subjected to LPS administration.

Temporal analysis of production performance parameters revealed patterns consistent with the three-phase stress response model. During days 14-16, LPS-induced stress precipitated decreased feed intake and body weight gain, representing the initial alarm phase characterized by stressor recognition and stress response mobilization. This phase typically exhibits reduced core body temperature, adrenergic activation, enhanced catabolism, and compromised performance and resistance capacity. Between days 16-20, the LPS treatment group exhibited significantly improved feed conversion efficiency compared to controls, despite no significant differences in feed intake or weight gain, indicating the adaptation and resistance phase. During days 20-22, LPS stress significantly reduced broiler weight gain, resulting in elevated feed conversion ratios, marking the exhaustion phase wherein prolonged stressor exposure compromises physiological function. At days 22-24, extended stress exposure activated compensatory mechanisms, resulting in significantly increased feed intake while weight gain and feed conversion ratio remained unchanged.

Corticosterone, the primary avian glucocorticoid, serves a critical regulatory role in environmental adaptation and stress response mechanisms. Under non-stressful conditions, broilers maintain baseline plasma corticosterone concentrations; however, stressor exposure rapidly elevates these levels. Previous investigations have demonstrated that immune stress increases corticosterone concentrations in broilers, inhibiting muscle protein synthesis while accelerating protein degradation (Li et al., 2019). The current study revealed significantly elevated plasma corticosterone levels at 4 and 8 hours post-initial LPS injection, with normalization occurring by 12 hours. Additionally, plasma corticosterone concentrations increased significantly 4 hours following the 4th and 6th LPS administrations. These findings indicate acute corticosterone elevation (0-8 hours) following LPS injection, confirming stress induction. In the hypothalamic-pituitary-adrenal axis (HPA axis), a central axis in stress regulation, poultry exhibit distinct dynamic patterns compared to rodents. Our studies have shown that broiler chickens display a biphasic corticosterone secretion pattern under LPS stimulation, characterized by a rapid increase in the early phase followed by a decline at 12 hours. In contrast, rodents such as mice exhibit a reduced cortisol response. This discrepancy may be attributed to the more pronounced regulation of poultry by the circadian rhythm of melatonin: photoperiod modulates hypothalamic CRH transcription via the pineal gland-suprachiasmatic nucleus axis, leading to stronger rhythmicity in their stress responses (Sun et al., 2023).

Li et al. demonstrated that vaccine-induced immune stress significantly increased bursal index in broilers, while Zhang showed that intraperitoneal LPS administration significantly decreased thymus index and notably increased spleen index (Li et al., 2020). Acute immune stress stimulates immune function and increases immune organ indices to meet heightened immune demands; however, prolonged stress without adequate nutritional support induces immune organ atrophy. In this investigation, LPS stimulation significantly increased spleen, thymus, and bursal indices, with substantially elevated splenic pro-inflammatory factor concentrations, indicating immune system activation.

In summary, continuous LPS stimulation decreased broiler performance parameters, elevated plasma corticosterone concentrations, and stimulated immune function, demonstrating that continuous intraperitoneal injection of 0.5 mg/kg LPS effectively established an immune stress model.

### Effect of immune stress on intestinal barrier function in broiler chickens

The ileum, positioned at the terminal small intestine, plays a significant role in immune regulation and neuroendocrine signaling, characterized by increased smooth muscle layer thickness and higher densities of lymphoid follicles and enteroendocrine cells relative to other intestinal segments. Consistent with previous findings, Li et al. demonstrated that immune stress compromises both immune barrier and physiological barrier functions in broilers (Li et al., 2024). However, the present study observed no significant alterations in ileal villus morphology, potentially due to insufficient stressor intensity to trigger negative morphological changes. Nevertheless, increased mRNA expression of pro-inflammatory cytokines TNF-α and IL-1β was observed, alongside decreased JAM2 gene expression involved in intestinal cell adhesion, indicating compromised intestinal physical and immune barrier integrity.

Additionally, immune stress reduced BCL2 and PCNA gene expression in broiler ileum. BCL2 functions as an anti-apoptotic gene, while PCNA serves as a cell proliferation marker (Zhang et al., 2018; Liang et al., 2017). Significant reduction in these gene expressions suggests that immune stress impacts intestinal cellular life processes. mTOR functions as a protein kinase regulating diverse cellular processes including growth, development, proliferation, and survival (Ma and Blenis, 2009). In vitro cellular assays demonstrate that mTOR inhibition affects intrinsic immune cell number and function (Weichhart et al., 2008), with mTOR signaling molecules regulating inflammatory factor transcription including IL-6 and IL-8 (Sun et al., 2015). Lorene et al. found that neutrophils activate mTOR phosphorylation during external stress (Lorne et al., 2009). The present study demonstrated significantly decreased mTOR gene expression with a tendency toward reduced P70S6K protein expression in ileum of immune-stressed broilers, suggesting that immune stress may trigger intestinal inflammatory factor release and regulate intestinal cell proliferation and differentiation life processes via the mTOR/P70S6K signaling pathway.

Collectively, these findings demonstrate that immune stress affects broiler intestinal barrier function, influencing cellular proliferation, differentiation, and apoptotic processes, with the mTOR signaling pathway potentially mediating these effects.

### Immune stress regulates growth performance and intestinal barrier function in broiler chickens via a neuro-endocrine network

Immune stress regulates broiler growth performance and intestinal barrier function through neuroendocrine network mechanisms. The neuroendocrine-immune network represents a well-established stress response pathway. Brain-gut connectivity occurs via a neuroendocrine network termed the brain-gut axis. During stress conditions, neuroendocrine chemical balance is disrupted. Adrenocorticotropic hormone and corticosterone, regulated by the hypothalamic-pituitary-adrenal (HPA) axis, and catecholamine neuropeptides (dopamine, epinephrine, and norepinephrine), regulated by the sympathetic-adrenal-medullary (SAM) axis, exhibit altered concentrations. Additionally, multiple hormones and brain-gut peptides coordinate to regulate animal physiological states and behaviors.

This investigation demonstrated that immune stress decreased NPY gene expression in broiler hypothalamus. NPY represents a key enteric nervous system neuropeptide with anti-stress properties; increased NPY release or receptor activation induces appetite enhancement and feeding behavior (Clark et al., 1984). Previous rat studies showed NPY decreases during chronic and acute restraint stress, with NPY promoting ingestive behaviors and food intake (Sun et al., 2015). However, immune stress in this study reduced NPY gene expression, ultimately suppressing broiler appetite.

MCH represents another neuropeptide regulating diverse neurophysiological functions including feeding, sleep-wake cycles, and emotional behaviors (Diniz and Bittencourt, 2019). Studies demonstrate MCH as a potent appetite-stimulating neuropeptide with central activity in rodents (Gomori et al., 2003). In the present study, hypothalamic MCH gene expression initially decreased four hours post-LPS injection, coinciding with appetite suppression. However, after 24 hours, MCH gene expression significantly increased, indicating LPS appetite-suppressing effect resolution, with the organism increasing MCH gene expression through self-regulation mechanisms.

The PREPRO gene produces orexin A and B neuropeptides involved in appetite and energy balance regulation (Tsujino and Sakurai, 2013). This study found immune stress significantly increased hypothalamic PREPRO gene expression, potentially representing a compensatory response to immune stress-induced orexin expression inhibition. Furthermore, LPS-treated broilers exhibited reduced feed intake at days 14-16 and tendency toward reduced intake at days 20-22, aligning with stress-induced hypothalamic appetite gene expression alterations. These findings suggest immune stress impacts hypothalamic neuropeptide synthesis, resulting in broiler appetite and behavioral changes, ultimately compromising performance and inducing abnormal behaviors.

Serotonin (5-HT) functions as a widely distributed neurotransmitter in central and peripheral nervous systems, with the majority produced by enterochromaffin (EC) cells. 5-HT plays a crucial role regulating gastrointestinal peristalsis and secretion, with abnormal increases leading to heightened visceral sensitivity. In the central nervous system, 5-HT regulates psychoaffective mood states. Upon gastrointestinal stimulation, EC cell-released 5-HT can directly modulate brain-gut axis chemical signals or bind to 5-HT receptors in intestinal mucosal epithelium, submucosa, or enteric plexuses, affecting gastrointestinal motility and visceral sensitivity. Under immune stress conditions, broiler serum 5-HT levels were significantly reduced, with significantly decreased ileal expression of 5-HT synthesis enzymes and receptor genes. However, no significant hypothalamic gene changes were observed, suggesting that intestinal 5-HT synthesis alterations are responsible for systemic 5-HT level changes, subsequently triggering gastrointestinal dysfunction.

In intestinal barrier function, avian responses also differ markedly from those of mammals. Reduced JAM2 gene expression in the broiler ileum suggests disruption of tight junctions, yet villus atrophy is absent a difference that may be related to the shorter length of the avian gut and its accelerated cell renewal. Unlike rodents, where serotonin (5-HT) synthesis relies primarily on enterochromaffin cells, avian 5-HT production is dominated by enteroendocrine-like cells (ECLs) (Saruhashi et al., 2009; Yu et al., 2024).

### Effect of immune stress on m^6^A methylation modification in broiler ileum and hypothalamus

The m^6^A represents the most prevalent RNA methylation modification, specifically and predominantly expressed in animal tissues with high expression in brain, liver, and kidney (Meyer et al., 2012). mRNA m^6^A methylation participates in virtually all RNA metabolic processes, playing crucial roles in physiological processes including growth and development, neural development, and immune responses. mRNA m^6^A methylation remains relatively stable, but its distribution can be influenced by environmental and genetic factors (Lv et al., 2018; He et al., 2017). As mRNA levels can change rapidly, this facilitates adaptation to sudden internal and external environmental changes. Research demonstrates that m^6^A modification-mediated stress responses can connect external stress to intracellular transcription and translation processes, indicating m^6^A methylation's significant role in animal stress responses.

The ileum and hypothalamus represent important secretory organs. Previous studies demonstrated that immune stress affects hypothalamic hormone secretion in broilers and reduces ileal endocrine and proliferative cell marker gene expression, suggesting immune stress weakens hypothalamic and ileal endocrine functions. In response to these changes, m^6^A methylation modifications increase, indicating that immune stress can regulate hypothalamic and ileal function by altering mRNA m^6^A methylation modifications in response to immune stress-induced organismal state changes.

The hypothalamus serves as a vital nerve center and endocrine organ. This study employed MeRIP-seq technology to identify m^6^A methylation modification profiles within broiler hypothalamic transcriptomes for the first time, aiming to understand molecular mechanisms of m^6^A methylation modifications in broiler immune stress responses. The investigation revealed that genes containing m^6^A methylation modifications in immune-stressed and control broilers were primarily enriched in metabolic and stress response regulatory pathways.

The Wnt signaling pathway is highly conserved, regulating cellular growth, development, and differentiation, playing crucial roles in normal organismal growth and development, particularly in nervous system function. In the central nervous system, Wnt signaling promotes adult hippocampal neurogenesis, synapse formation, and enhances neuronal plasticity and neurotransmission (Farias et al., 2010). The ErbB signaling pathway participates in neural synapse growth, neuronal migration, and differentiation, essential for proper central nervous system function (Hynes and MacDonald, 2009). The mTOR signaling pathway participates in protein, nucleotide, and lipid synthesis and metabolism, regulating cellular proliferation and apoptosis through autophagy inhibition. Immune stress triggers immune responses including MAPK signaling pathway-induced cytokine transcription.

Following immune stress exposure, broilers experience transcriptomic m^6^A methylation modification redistribution, resulting in altered m^6^A modifications in genes related to hypothalamic neurodevelopment and metabolic processes. However, further investigation is required to elucidate specific underlying mechanisms.

## Conclusion

Collectively, these findings demonstrate that sustained LPS stimulation induces immune stress, which inhibits the mTOR/p70S6K signaling pathway in broiler ileum. This inhibition significantly impacts critical cellular processes, including differentiation and proliferation pathways. Consequently, this leads to a reduction in secretory cell populations and a concomitant increase in absorptive cell populations within the ileal epithelium. Immune stress also modulates intestinal 5-HT secretion and receptor expression profiles, induces mucosal inflammatory responses, and compromises intestinal barrier integrity. Moreover, immune stress regulates hypothalamic neurotransmitter expression and their cognate receptors, as well as metabolic processes, through alterations in hypothalamic RNA m^6^A methylation modifications. Additionally, immune stress suppresses hypothalamic appetite-regulatory gene expression, ultimately resulting in compromised production performance parameters.

## Ethics approval and consent to participate

All animal care and use procedures were evaluated and approved by the Animal Care and Experiment Committee of China agricultural university.

## Availability of data and material

The datasets and materials used in this study are available from the corresponding author upon reasonable request. All data supporting the conclusions of this article are included within the article.

## Consent for publication

Not applicable.

## CRediT authorship contribution statement

**Bingjian Huang:** Conceptualization, Methodology, Writing – original draft. **Yan Wan:** Investigation, Methodology. **Guang Li:** Methodology, Supervision. **Jian Cui:** Methodology. **Qiuyu Jiang:** Conceptualization, Methodology. **Xiang Zhong:** Methodology, Investigation. **Bingkun Zhang:** Methodology, Supervision.

## Declaration of competing interest

I hereby declare that there are no financial or personal relationships that could inappropriately influence or bias the objectivity or integrity of the research described in this article. This study has been approved by the corresponding author. This research was financially supported by the National Natural Science Foundation of China (No. 32072750), the China Agricultural Research System (CARS-41-G11), and the 2115 Talent Development Program of China Agricultural University.
